# Next-generation DNA barcoding: using next-generation sequencing to enhance and accelerate DNA barcode capture from single specimens

**DOI:** 10.1111/1755-0998.12236

**Published:** 2014-02-19

**Authors:** Shadi Shokralla, Joel F Gibson, Hamid Nikbakht, Daniel H Janzen, Winnie Hallwachs, Mehrdad Hajibabaei

**Affiliations:** * Department of Integrative Biology, Biodiversity Institute of Ontario, University of Guelph50 Stone Road East, Guelph, ON, Canada, N1G 2W1; † Department of Microbiology, Mansoura UniversityEgypt, 35516; ‡ Department of Biology, University of PennsylvaniaPhiladelphia, PA, 19104, USA

**Keywords:** biodiversity, *
COI
*, DNA, genomics, heteroplasmy, Lepidoptera, taxonomy, *Wolbachia*

## Abstract

DNA barcoding is an efficient method to identify specimens and to detect undescribed/cryptic species. Sanger sequencing of individual specimens is the standard approach in generating large-scale DNA barcode libraries and identifying unknowns. However, the Sanger sequencing technology is, in some respects, inferior to next-generation sequencers, which are capable of producing millions of sequence reads simultaneously. Additionally, direct Sanger sequencing of DNA barcode amplicons, as practiced in most DNA barcoding procedures, is hampered by the need for relatively high-target amplicon yield, coamplification of nuclear mitochondrial pseudogenes, confusion with sequences from intracellular endosymbiotic bacteria (e.g. *Wolbachia*) and instances of intraindividual variability (i.e. heteroplasmy). Any of these situations can lead to failed Sanger sequencing attempts or ambiguity of the generated DNA barcodes. Here, we demonstrate the potential application of next-generation sequencing platforms for parallel acquisition of DNA barcode sequences from hundreds of specimens simultaneously. To facilitate retrieval of sequences obtained from individual specimens, we tag individual specimens during PCR amplification using unique 10-mer oligonucleotides attached to DNA barcoding PCR primers. We employ 454 pyrosequencing to recover full-length DNA barcodes of 190 specimens using 12.5% capacity of a 454 sequencing run (i.e. two lanes of a 16 lane run). We obtained an average of 143 sequence reads for each individual specimen. The sequences produced are full-length DNA barcodes for all but one of the included specimens. In a subset of samples, we also detected *Wolbachia*, nontarget species, and heteroplasmic sequences. Next-generation sequencing is of great value because of its protocol simplicity, greatly reduced cost per barcode read, faster throughout and added information content.

## Introduction

The use of short, standardized DNA sequences, or DNA barcodes, for the purposes of individual identification of organisms has contributed to different areas of biological research (Savolainen *et al*. 2005; Hajibabaei *et al*. 2007). DNA barcodes can be used to detect undescribed and cryptic species (e.g. Hebert *et al*. 2004; Janzen *et al*. 2011, 2012; Chacon *et al*. 2013), to allow complex ecological interactions to be investigated (e.g. Smith *et al*. 2006, 2011) and to determine accuracy of species content of commercial products (e.g. Wallace *et al*. 2012). The mitochondrial protein-coding gene cytochrome *c* oxidase subunit I (*COI*) has been established as the standard DNA barcode for the identification of animals (Hebert *et al*. 2003; Hajibabaei 2012). The nuclear internal transcribed spacer (*ITS*) ribosomal DNA gene region is the comparable DNA barcode for fungi (Schoch *et al*. 2012), while the *rbcL* and *matK* gene regions are used as DNA barcodes for plants (Hollingsworth *et al*. 2009). The building and curation of libraries of DNA barcodes for the entirety of living organisms is the focus of a number of international efforts (e.g. Savolainen *et al*. 2005; Hajibabaei *et al*. 2007). From the beginning of such efforts until today, the need to increase both the taxonomic coverage of species included in such databases as well as the pace at which species are added has been strongly emphasized (Hajibabaei *et al*. 2005; Kwong *et al*. 2012; Kvist 2013).

The conventional means of generating DNA sequence data to obtain a barcode for a species or a specimen are through PCR amplification and Sanger sequencing (Sanger *et al*. 1977) of DNA barcode sequences from genomic DNA extracted from individual specimens. For library construction, this is for well-identified specimens, while for species detection, the specimen need not be described. Sanger sequencing technology is capable of generating sequencing reads of up to 1000 bases and was the only approach used for DNA sequencing for nearly three decades, but next-generation sequencing (NGS) devices are now beginning to dominate the sequencing market (see below). Consequently, many genomics centres have phased out their Sanger sequencers and have switched to NGS. Aside from low throughput, Sanger sequencing requires a DNA amplicon template of high concentration (100–500 ng) to avoid inherent biases and errors (Polz & Cavanaugh 1998). In addition, Sanger sequencing provides a single sequencing signal pattern, or electropherogram, for each sequence generated. Preferential or coamplification of nonbarcode sequences such as pseudogenes or intracellular, endosymbiotic bacteria (e.g. *Wolbachia*) can occur during the PCR amplification step (e.g. Song *et al*. 2008; Smith *et al*. 2012), leading to confusion of identity among the ‘true’ barcode and other sequences. Likewise, a number of studies have revealed instances of intraindividual mitochondrial variability, or heteroplasmy, within a single animal (e.g. Matsumoto 2003; Magnacca & Brown 2010; Berthier *et al*. 2011; Vollmer *et al*. 2011). Additionally, the fungal DNA barcode, ITS, has been shown to be present as multiple variable copies within an individual’s genome (Schoch *et al*. 2012), thus leading to difficulty in direct Sanger sequencing of amplicons. Any or all of these situations can lead to ambiguity or false information in generated DNA barcodes when the Sanger sequencing method is employed, and may result in repeated or failed sequencing attempts. In the best-case scenario, competing signals are so weak that they do not influence the generation of a single barcode. In these cases, however, the genetic information contained within these alternate DNA sequences is discarded to generate a single barcode sequence.

Next-generation sequencing (NGS) technologies (reviewed in Shokralla *et al*. 2012) allow for the sequencing of millions of DNA fragments, from thousands of DNA templates in parallel. 454 pyrosequencing was the NGS platform first to be introduced to the market (Margulies *et al*. 2005). In its current incarnation, it can generate up to one million DNA sequences up to 700 bases in length in a single sequencing run. The platform was developed and is still mainly used to obtain DNA sequence information of whole genomes or from bulk environmental samples. However, methods have also been developed to individually tag amplicons (using a set of oligonucleotides with a known sequence), combine them into a single sequencing run and recover them bioinformatically (Binladen *et al*. 2007). For this reason, NGS technology could be employed to overcome some of the inherent limitations of Sanger-based sequencing for DNA barcoding efforts. This new approach could also potentially facilitate the generation of DNA barcodes more quickly and at a lower total cost by taking advantage of the higher throughput offered by NGS technologies.

In the present study, we apply an NGS approach to the parallel acquisition of DNA barcode sequences from 190 specimens simultaneously. We used specifically designed 10-mer oligonucleotide tags attached to DNA barcoding PCR primers to uniquely tag amplicons. The specimens are Lepidoptera species collected as a part of an ongoing inventory of the biodiversity of Area de Conservación Guanacaste (ACG), northwestern Costa Rica (Janzen *et al*. 2009, 2011, 2012). We perform a side-by-side comparison of the traditional Sanger sequencing versus 454 pyrosequencing in generating DNA barcodes for specimens of Lepidoptera to census the performance of an NGS approach. We also assess whether an NGS approach is capable of overcoming the limitations of Sanger sequencing. We report sequence data from the possible causes of Sanger sequencing failure – degraded or low-concentration DNA template; nontarget contamination; heteroplasmy; and intracellular bacteria.

## Materials and methods

### DNA extraction, PCR amplification and Sanger sequencing

A total of 190 individual specimens of Lepidoptera from Area de Conservación Guanacaste, northwestern Costa Rica, were selected for inclusion. This sample included 106 specimens of Nymphalidae and 84 specimens representing thirteen other families of Lepidoptera. Two sets of specimens were tested in this study: one set of specimens [95 samples (Lane 1)] was of reared Lepidoptera collected specifically for this study (Table S1, Supporting information) and a second set of 95 samples (Lane 2) included a set of reared voucher specimens collected for routine DNA barcoding (Sanger sequencing) (Table S2, Supporting information). Please see supplementary material for detailed collection data. Tissue subsamples (i.e. 2–4 mm of leg tissue) from each individual were DNA extracted using a Nucleospin® Tissue kit (Macherey-Nagel Inc., Bethlehem, PA, USA) according to manufacturer’s protocols.

The standard DNA barcode region at 5′ end of the *COI* gene was amplified using the primers LepF1 and LepR1 (Hebert *et al*. 2004). PCR reactions were assembled in a 25 *μ*L volume [2 *μ*L DNA template, 17.5 *μ*L molecular biology grade H_2_O, 2.5 *μ*L 10× Invitrogen buffer, 1 *μ*L 50× MgCl_2_ (50 mm), 0.5 *μ*L dNTPs mix (10 mm), 0.5 *μ*L forward primer (10 *μ*m), 0.5 *μ*L reverse primer (10 *μ*m) and 0.5 *μ*L Invitrogen Platinum Taq polymerase (5 U/*μ*L)]. The amplification cycle was: 95 °C for 5 min; 35 cycles of 94 °C for 40 s, 51 °C for 1 min, 72 °C for 30 s; final extension at 72 °C for 5 min. All amplifications were completed on a Mastercycler ep gradient S (Eppendorf, Mississauga, ON, Canada). A negative control reaction with no DNA template was included in all experiments. All generated amplicons were visualized on 2% E-gels 96 Agarose (Invitrogen, Burlington, ON, Canada) to measure PCR success. The amplicons generated from all 190 specimens were also individually Sanger sequenced using an ABI 3730XL sequencer (Applied Biosystems, Foster City, CA, USA).

### Tag design and 454 pyrosequencing

**Figure 1:**
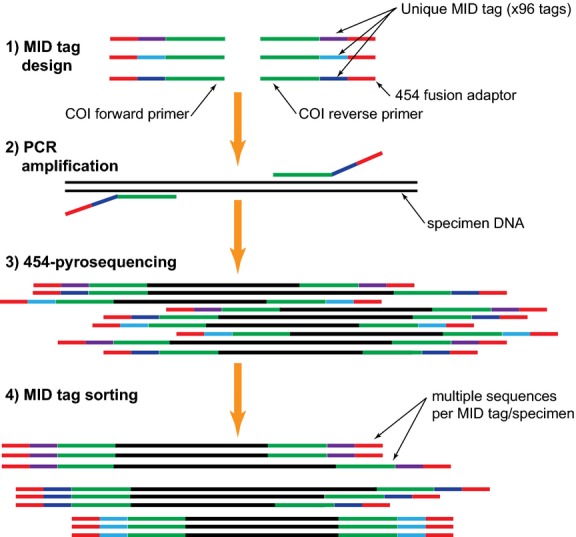
Schematic diagram of parallel barcode recovery using multiple identifier (MID) tagging and next-generation sequencing (NGS) protocol.

A total of 96 unique 10-mer multiple identifiers (MIDs) were designed (Table S3, Supporting information). Rather than random nucleotide sequences, five key factors were considered during MID tag design: (i) MID tags could not begin or end with the same nucleotide as the 454 Titanium fusion adaptor sequence; (ii) MID tags could not begin or end with the same nucleotide as the PCR amplification primer; (iii) homopolymers of greater than two nucleotides were not allowed; (iv) MID tags must differ from one another by at least two nucleotides; and (v) the successive positive incorporation of two nucleotides during the TCAG cycle was avoided. Custom primers were ordered that included either the LepF1 or LepR1 PCR amplification sequence, an MID tag, or the 454 Titanium fusion sequence (Fig.1).

DNA templates from all 190 specimens were PCR amplified with the tagged and fusion-tailed primers in the same reaction conditions as above. To ensure a homogeneous number of sequencing reads from each specimen, amplicons were mixed in equal concentrations (100 pg/*μ*L) in an equimolar pool. Amplicons, primer dimers and PCR artefacts of ≤100 bp were eliminated by size selection with AMPure magnetic beads (Invitrogen, Burlington, ON, Canada). Purified libraries were quantified with a TBS 380 Mini Fluorometer (Turner BioSystems, Promega, USA) and visualized on a DNA chip in an Agilent 2100 Bioanalyzer (Agilent Technologies, USA). Quantified, size-selected amplicon libraries (779 bp including amplification primers, MID tags and 454 adaptors) were serially diluted to several final concentrations to give the optimal copy per bead ratio through the amplification step according to the GS FLX amplicon library preparation protocol. Emulsion PCR, bead recovery and enrichment steps were performed using a GS FLX emPCR amplicon kit according to the manufacturer’s protocols. The amplified, enriched beads from each of two libraries (95 specimens each) were sequenced in two physically separated lanes (1/16) of a standard PicoTiter plate (70 × 75) using the standard GS Titanium sequencing kit XLR 70 (454 Life Sciences, Branford, CT, USA) according to the manufacturer’s protocols.

### Bioinformatic analysis

Sanger-generated sequences were edited and assembled using codoncode aligner v3.7.1.1 (CodonCode Corp., Dedham, MA, USA). All 454 pyrosequencing sequences were trimmed from the 3′ end [Phred quality threshold (Q) = 20; window size = 10, sliding step = 5] using prinseq (Schmieder & Edwards 2011). All trimmed sequences were sorted by MID tag allowing zero mismatches in the tag sequence. Amplification primers and MID tags were then removed. Sequences within each MID tag were clustered using uclust (Edgar 2010) at 100% similarity. Both forward and reverse clusters were used to assemble contigs using mega v.5.2.2 (Tamura *et al*. 2011).

All Sanger-generated sequences and 454 pyrosequencing sequence clusters >600 bp in length were searched against a locally stored database of *COI* barcode sequences previously determined in barcoding ACG specimens (retrieved from BOLD and GenBank) using megablast (Zhang *et al*. 2000) at 98% similarity for identifications. To further verify the matches, recovered sequences were aligned and pairwise distance analysis was performed using mega v.5.2.2 (Tamura *et al*. 2011). Details of Sanger sequencing and 454 NGS sequence data are available in Tables S1 and S2 (Supporting information). All 454 pyrosequences and assembled contigs have been deposited in GenBank (accession nos: KJ101612–KJ101695; KJ101696–KJ111585; KJ111586–KJ111678; and KJ111679–KJ123638) and DRYAD entry: doi:10.5061/dryad.p25vn.

## Results

For 178 of the 190 specimens (93.7%), identifiable DNA sequences were recovered by the Sanger method. Of these 178 sequences, 127 (71.3%) were of the full 658 bp barcode length. For 189 of the 190 specimens (99.5%), at least one identifiable 658 bp sequence was recovered by the 454 pyrosequencing method. All 189 sequences were of the full 658 bp barcode length. The identified specimens represented fourteen families and 56 genera of Lepidoptera. Of the twelve specimens for which a Sanger sequence was not recovered, four specimens (33%) recovered more than one distinct 454 sequence.

**Figure 2:**
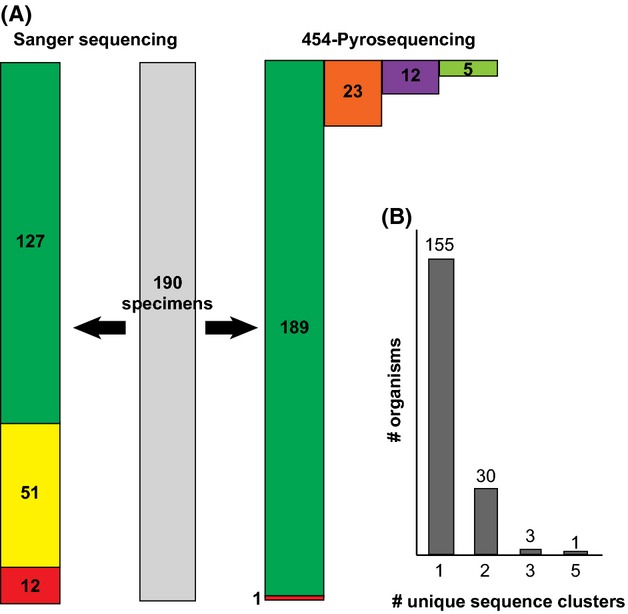
Comparison of DNA sequence data recovered by Sanger sequencing and 454 pyrosequencing. A) Green bars represent number of full-length *COI* barcode sequences. Yellow bar represents number of partial *COI* barcode sequences. Red bars represent failed target barcode attempts. Orange bar represents number of heteroplasmic *COI* sequences. Purple bar represents number of coamplified nontarget *COI* sequences (i.e. ‘contaminants’). Light green bar represents number of *Wolbachia* sequences. B) Number of organisms recovering single or multiple sequence clusters during 454 pyrosequencing.

A total of 34 170 sequences (19 909 sequences for Lane 1 and 14 261 sequences for Lane 2) were recovered through 454 pyrosequencing. The length of sequences ranged from 40 to 927 bp. Following quality filtering, MID tag sorting and trimming, 26 281 sequences remained. An average of 143 sequences (minimum 40, maximum 259) was assigned to each MID tag in each lane. Each MID tag represented a single specimen and recovered between one and five distinct DNA sequences (Fig.2). Filtered and sorted sequences were clustered into a total of 229 sequence clusters. Of these, 189 (82.5%) represented a single, exactly identifiable, 658 bp barcode sequence matching a sequence in the Sanger library (where available in the library). Five (2.1%) sequences from five specimens were identified as *Wolbachia* symbionts. Twelve (5.2%) sequences from ten specimens were identified as animal *COI* sequences not matching the corresponding Sanger-recovered barcode identity. Eleven of these sequences from nine specimens matched the identity of another Lepidoptera species collected at the same time. The final sequence, out of the twelve sequences, matched *Homo sapiens*. Each of these sequences is likely the result of cross-contamination of specimens during field collection or tissue subsampling. The remaining 23 sequences (10%) differed from another sequence cluster with the same MID tag but matched the same species with 98% similarity. These sequences were identified as likely heteroplasmic sequences. The similarity between these heteroplasmic sequences and their corresponding sequences with the same MID tag was greater than 98%. Of the 34 MID tags that produced greater than one sequence cluster, eight (23.5%) produced one sequence that represented at least 90% of the sequences produced (Fig.2).

**Figure 3:**
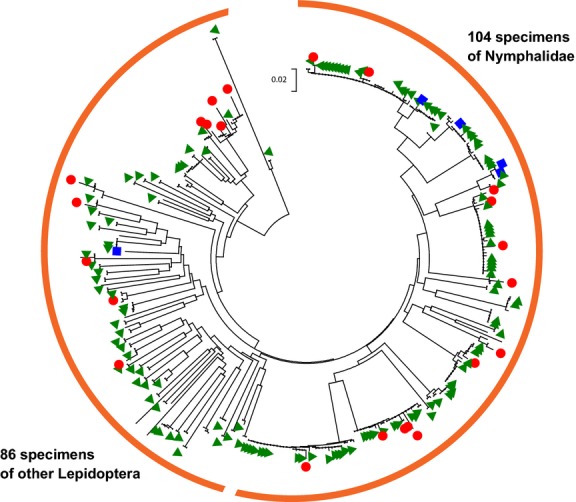
Neighbour-joining diagram of 352 DNA sequences recovered by 454 pyrosequencing and Sanger sequencing. Short sequences (<600 bp) have not been included. Distance measurement is calculated in number of base substitutions per site based on the Kimura 2-parameter method. The tree backbone represents the 454 pyrosequences and green triangles represent sequences produced by Sanger sequencing (>600 bp). Red circles represent sequences determined to be heteroplasmic. Blue squares represent individual specimens that also recovered a *Wolbachia* sequence.

A Neighbour-joining analysis based on Kimura 2-parameter distance (Kimura 1980) recovered all Sanger produced sequences >600 bp within a node containing the associated 454 pyrosequencing targets in addition to all 454 pyrosequences identified as heteroplasmic variants (*n* = 352; Fig.3).

## Discussion

**Figure 4:**
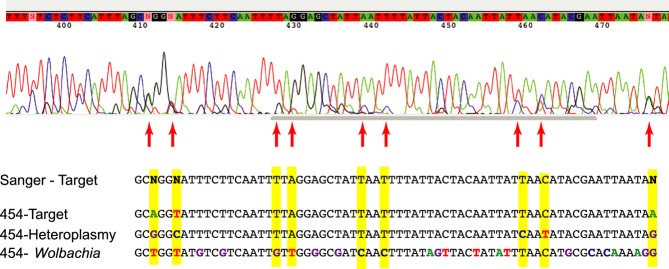
Portion of a sequence electropherogram as produced by Sanger sequencing and composite sequence clusters as recovered by 454 pyrosequencing of a single specimen. Highlighted bases represent differences from the Sanger sequence. Arrows indicate the presence of peaks in the electropherogram corresponding to alternate sequences.

Sanger sequencing has been the dominant approach for obtaining DNA sequence information from single specimens in a wide range of applications from molecular diagnostics to ecological and taxonomic analysis. In many applications, such as DNA barcoding using a mitochondrial gene in animal species, a presumably homogenous amplicon can provide a high-quality Sanger sequence read which corresponds to the target DNA barcode. However, traditionally, a cloning step had been added to Sanger sequencing workflow to obtain sequence information from multiple clones to investigate potential coamplification (e.g. Song *et al*. 2008). Coamplification of nontarget sequences may result in the failure of Sanger sequencing to recover DNA barcodes (Song *et al*. 2008; Boessenkool *et al*. 2012). In the instance of coamplification, multiple sequences from the same specimen are amplified and are shown as overlapping signals in a Sanger sequencing electropherogram (Fig.4). Of the twelve specimens for which a Sanger sequence was not recovered, eight of them produced a single 454 pyrosequencing cluster. These instances were likely examples of specimens with low amplicon yield that were only recoverable due to the increased sensitivity of the NGS approach. One specimen produced neither a Sanger barcode nor a 454 pyrosequencing cluster. This specimen could possibly have been degraded beyond the possibility of any DNA recovery. The remaining three specimens all recovered multiple pyrosequencing clusters. In addition to the target sequence, these specimens also produced sequences from *Wolbachia* or nontarget contaminants (Fig.2). In these cases, it is likely that the presence of competing, nontarget sequence prevented the successful recovery of a single DNA barcoding sequence using the Sanger approach. This observation echoes others (e.g. Song *et al*. 2008; Smith *et al*. 2012) that have suggested that the presence of *Wolbachia* or heteroplasmy in public *COI* sequence databases could compromise attempts at DNA barcode specimen identification.

Beyond the use of DNA barcodes to identify specimens, the approach is also used to add new species records to public barcode libraries. Based on our NGS data, in most cases, it is possible to assign a single, consensus sequence as a reference DNA barcode for an individual specimen. For 155 of the specimens studied, a single consensus DNA sequence was generated using the 454 pyrosequencing approach (Fig.2). A further eight specimens produced a single consensus sequence that represented over 90% of the sequences produced with a single MID tag. In these instances, this single, abundant sequence is most likely the DNA barcode, and the other sequences may represent contamination, endosymbiotic bacteria or heteroplasmy.

Of the 34 MID tags that produced greater than one sequence cluster, eight (23.5%) produced one sequence that represented at least 90% of the sequences produced. For the remaining 26 specimens, multiple sequences are recovered, each representing less than 50% of the sequences produced. The possibility of heteroplasmy can be checked by examining the similarity between the sequences. In the examples of heteroplasmy included here, the sequences differed by less than 2% overall similarity and both sequences can be recorded as the DNA barcode for the specimen. By utilizing the parallel sequence capabilities of NGS approaches, more light can be brought to instances of single outlying sequences within well-defined species clusters based on DNA barcodes (e.g. Janzen *et al*. 2011). In these cases, cryptic diversity may not be the answer, but instead the presence of a competing signal from heteroplasmic sequences.

Mitochondria are assumed to be almost exclusively maternally inherited and do not mutate within an individual. As such, there is assumed to be only a single haplotype of each mitochondrial gene region within any given organism. A number of cases in the past, however, have demonstrated the presence of more than one version of a mitochondrial gene region within an individual (e.g. Matsumoto 2003; Magnacca & Brown 2010; Berthier *et al*. 2011; Vollmer *et al*. 2011). As many as 59% of individuals within some species may be heteroplasmic for a certain gene region (Vollmer *et al*. 2011). Furthermore, it has been suggested that heteroplasmy within the *COI* gene region could compromise attempts to DNA barcode some insect species (Brower 2006; Magnacca & Brown 2010).

The difference between the 23 instances of presumed heteroplasmic sequences in this study was less than 2% overall similarity. There was no evident phylogenetic pattern to the presence of heteroplasmy among Lepidoptera as fourteen different species, eleven different genera and four different families were represented amongst individuals demonstrating heteroplasmy (Fig.3). The presence of heteroplasmy was also not universal to any species, as individuals producing heteroplasmies and those not were both represented in all cases where multiple specimens were included. Recombination, paternal leakage and biparental inheritance have all been offered as explanations for this phenomenon (Kondo *et al*. 1990; Vollmer *et al*. 2011). The presence of heteroplasmy and mutations within the individual may be of extreme importance for those studying the biogeography or evolutionary history of a species or population. For these reasons, reporting any possible heteroplasmy/mutations should be an important component of barcoding efforts. The NGS-based DNA barcoding approach, as shown in our analysis, is capable of recovering sequence variation in amplicons obtained from each specimen (Taylor & Turnbull 2005; Magnacca & Brown 2010).

The alpha-proteobacterial genus, *Wolbachia*, contains a number of species that have been isolated from inside the cells of arthropods. These endosymbionts have been demonstrated to induce feminization, parthenogenesis, male killing and cytoplasmic incompatibility in a number of different insect orders (reviewed in Engelstädter & Hurst 2009). A recent survey (Smith *et al*. 2012) of the presence of bacterial endosymbiotic DNA sequences within insect DNA barcode libraries found that while overall numbers of *Wolbachia* sequences were low (0.16%), they differed greatly amongst insect orders. Smith *et al*. (2012) concluded that this low overall rate is not likely to compromise large-scale DNA barcoding efforts of multiple specimens per species. In the present study, five instances of *Wolbachia* infection were detected in three different species of Lepidoptera (Fig.3). For one species, *Opisphanes tamarindi* (Nymphalidae), both specimens tested recovered a *Wolbachia* as well as the target DNA barcode sequence. For the second species, *Caligo telamonius* (Nymphalidae), many individuals were sampled that did show evidence of *Wolbachia* infection, and this had already created the suspicion of either *Wolbachia* or two sibling sympatric species of *C. telamonius*. The two specimens of *O. tamarindi* that did recover *Wolbachia* sequences recovered two different species of the endosymbiont. For the final individual recovering a *Wolbachia* sequence (Hesperiidae: *Carystus phorcus*), only a single individual was included in our sampling.

While it is not likely for *Wolbachia* DNA sequences to be confused with those of their insect hosts (Fig.4), the simultaneous recording of both bacteria and insect DNA sequences in the same individuals may be of high scientific value. A number of recent studies (e.g. Pan *et al*. 2012; Hoffmann & Turelli 2013) have investigated the effects of wild and introduced strains of *Wolbachia* on the disease transmission capabilities of mosquitoes. Other research has investigated the impact of strains of *Wolbachia* on the evolutionary history of wild populations of insects (e.g. Dyer & Jaenike 2004). In these instances, and others, the simultaneous recovery of both host DNA and *Wolbachia* DNA would be of major benefit to research efforts.

The presence of DNA sequence signal from a low-concentration competing source can often cause Sanger-based sequencing of an organism to fail to generate the actual DNA barcode. A number of standard protocols are in place to reduce or eliminate the risk of cross-contamination during DNA extraction, PCR amplification and sequencing (Hajibabaei *et al*. 2005; Boessenkool *et al*. 2012). Even with such measures in place, some sources of nontarget contamination are unavoidable, as was the case with the examples in the ACG Nymphalidae and Hesperiidae mentioned above. Organisms that are caught in bulk and stored in a medium (e.g. ethanol) will experience dispersion of DNA throughout the preservative fluid (Shokralla *et al*. 2010; Hajibabaei *et al*. 2012). Insect hairs and scales can also be unnoticeably transferred from specimen to specimen prior to tissue subsampling and individual DNA barcoding (Janzen *et al*. 2009). In addition to cross-contamination, tissues of many organisms can contain protists (e.g. Týč *et al*. 2013), nematodes (Park 2012) or mites (Knee *et al*. 2012) that can be recovered during DNA barcoding. If an entire specimen is sampled, it may also contain larvae and eggs of parasitoid insects (e.g. wasps, flies) or gut contents from entomophagy. Any of these sources can introduce competing, nontarget DNA sequence data. While contamination should continue to be avoided in any laboratory setting, our protocol prevents low-concentration, nontarget DNA from compromising attempts to recover the DNA barcode for a target individual. The DNA sequence from the contamination source will often be recovered with a much lower number of sequences. In those cases where the single, most abundant DNA sequence cannot be determined through sequence analysis, information from morphological analysis may be necessary to determine the ‘true’ DNA barcode. That is to say, for example, if the target specimen was *Carystus* in the Hesperiidae, then the ‘true’ barcode needs to be similar to that of other *Carystus* in the Hesperiidae.

## Conclusion

By utilizing MID tagging, NGS 454 pyrosequencing and bioinformatic recovery, the DNA barcodes of 190 specimens were recovered using 1/8th of a complete 454 pyrosequencing run with greater success as compared to conventional Sanger-based sequencing. Next-generation sequencing devices provide significantly lower cost per base as compared to Sanger sequencing; hence, efficient use of MID tagging can result in more cost-effective DNA barcode analysis. Additional sequence information (e.g. heteroplasmy, contamination, *Wolbachia*) gained from parallel sequencing of each specimen, through NGS, is a unique feature that cannot be feasibly achieved through Sanger sequencing. The error rate of 454 pyrosequencing requires the exclusion, through bioinformatic filters, of a large number of the sequences produced. While base substitution is a rare occurrence, the most common pyrosequencing error in sequencing by synthesis with multiple nucleotide incorporation is the over or under base calling especially in homopolymeric regions (Margulies *et al*. 2005). Several bioinformatic solutions are available to deal with such sequencing artefacts (e.g. Gilles *et al*. 2011). For example, screening for nucleotides with low Phred scores within homopolymeric regions and using the amino acid reading frame in protein-coding markers can further overcome the negative impact of this issue (Shokralla *et al*. 2011). Moreover, increased sequencing read length and depth can statistically alleviate the effect of the base calling errors. Both of these factors can contribute to a higher probability of obtaining high-quality sequences in the output sequences generated. In this respect, newer versions of 454 pyrosequencing or other NGS platforms have improved their sequencing output to decrease the probability of sequencing errors (Taberlet *et al*. 2012).

The total number of sequences produced limits the number of specimens that can be included in a run if sufficient sequencing depth is to be maintained. A decreased error rate and increased sequencing throughput will further amplify the rate at which DNA barcodes can be produced with NGS technology. Our study provides a new example of the application of NGS in a realistic high-throughput DNA barcoding scenario and sets the stage for further use of NGS devices in routine single specimen DNA barcoding. Although we have tested our approach on a Roche-454 FLX model, the technique is not platform-specific and can be applied to other available NGS platforms. For example, desktop NGS devices (e.g. Illumina MiSeq, Roche-454 Junior, Ion Torrent PGM) are now feasible options for any laboratory (Table1). However, prior to NGS-based DNA barcoding efforts, MID tags must be tested for different NGS platforms according to the specific chemistry and the sequencing errors associated with each.

**Table 1:** General specifications of the most commonly used next-generation sequencing (NGS) platforms as compared to Sanger sequencing

	454 GS FLX +	454 Junior	Illumina HiSeq	Illumina MiSeq	Ion Torrent PGM	Sanger ABI 3730xl
Max. read length	700 bp	400 bp	2 × 150 bp	2 × 300 bp	400 bp	1–1.5 kb
Max. output/run	450–700 Mb	35 Mb	150–180 Gb	13.2–15 Gb	1.2–2 Gb	96 Kb
Max. reads/run	700 k–1 M S-R	70 K S-R	1.2 Billion PE-R	44–50 M PE-R	4–5.5 M S-R	96 S-R
Time per run	23 h	10 h	40 h	65 h	7.3 h	4 h

S-R, single reads; PE-R, paired-end reads; Information in this table was obtained from manufacturers’ web pages accessed on 7 January 2014.

The number of specimens to be multiplexed in a single experiment depends on at least four factors: (i) the number of MID tags compatible with the adaptor sequences of each platform; (ii) the possibility of sequencing the same MID tags in physically separated lanes of a single run; (iii) the number of generated sequences per run; and (iv) the required sequence depth needed per specimen. A single NGS device can be used for a wide range of applications from genome and transcriptome sequencing to environmental metagenomics and DNA metasystematics (Hajibabaei *et al*. 2012; Shokralla *et al*. 2012; Taberlet *et al*. 2012). Here, we demonstrate the feasibility of NGS for single specimen DNA barcoding for library preparation or specimen identification. The application of NGS in specimen barcoding, however, should require standardized and rigorous platform-specific quality control steps to ensure the highest-quality DNA barcodes. When millions of DNA strands can be sequenced in parallel and many hundreds can be assigned to each component target amplicon, there is no need to generate a single DNA sequence.

## Notes

S.S. and M.H. conceived and designed the experiments. D.H.J. and W.H. collected all specimens examined. S.S. designed all MID tags and performed Sanger sequencing and 454 pyrosequencing. S.S., J.F.G., H.N. and M.H. analysed sequence data. S.S., J.F.G., D.H.J., W.H. and M.H. wrote and edited the final manuscript.

## Data Accessibility

DNA sequences: All sequence data have been deposited into GenBank (accession nos: KJ101612 – KJ101695; KJ101696 – KJ111585; KJ111586 – KJ111678; and KJ111679 – KJ123638) and DRYAD entry: doi:10.5061/dryad.p25vn.

## Supporting Information

Table S1 Sanger and 454 pyrosequencing data for specimens included in Lane 1.

Table S2 Sanger and 454 pyrosequencing data for specimens included in Lane 2.

Table S3 96 newly designed 10-mer MID tag sequences used for 454 pyrosequencing.
